# Conserved topology of virus glycoepitopes presents novel targets for repurposing HIV antibody 2G12

**DOI:** 10.1038/s41598-022-06157-z

**Published:** 2022-02-16

**Authors:** Nathaniel L. Miller, Vidya Subramanian, Thomas Clark, Rahul Raman, Ram Sasisekharan

**Affiliations:** 1 Department of Biological Engineering, Massachusetts Institute of Technology, Cambridge, MA 02139 USA; 2 Koch Institute for Integrative Cancer Research, Massachusetts Institute of Technology, Cambridge, MA 02139 USA; 3 Harvard-MIT Division of Health Sciences and Technology, Massachusetts Institute of Technology, Cambridge, MA 02139 USA; 4 Singapore-MIT Alliance in Research and Technology (SMART), Singapore, 138602 Singapore

**Keywords:** Drug discovery, Structural biology

## Abstract

Complex glycans decorate viral surface proteins and play a critical role in virus–host interactions. Viral surface glycans shield vulnerable protein epitopes from host immunity yet can also present distinct “glycoepitopes” that can be targeted by host antibodies such as the potent anti-HIV antibody 2G12 that binds high-mannose glycans on gp120. Two recent publications demonstrate 2G12 binding to high mannose glycans on SARS-CoV-2 and select Influenza A (Flu) H3N2 viruses. Previously, our lab observed 2G12 binding and functional inhibition of a range of Flu viruses that include H3N2 and H1N1 lineages. In this manuscript, we present these data alongside structural analyses to offer an expanded picture of 2G12-Flu interactions. Further, based on the remarkable breadth of 2G12 N-glycan recognition and the structural factors promoting glycoprotein oligomannosylation, we hypothesize that 2G12 glycoepitopes can be defined from protein structure alone according to N-glycan *site* topology. We develop a model describing 2G12 glycoepitopes based on N-glycan site topology, and apply the model to identify viruses within the Protein Data Bank presenting putative 2G12 glycoepitopes for 2G12 repurposing toward analytical, diagnostic, and therapeutic applications.

## Introduction

The epitope surfaces on antigens that are targeted by an antibody have largely been defined based on amino acid contacts between the antibody and antigen. The surface of a virus presents epitopes of varying complexities ranging from a defined region on a single protein domain to interfaces involving several copies of the surface protein along different axes of symmetry in the entire viral assembly^1,2^. In this context, many viral surface antigens are also heavily glycosylated by the host’s glycosylation machinery and glycans displayed on the viral surface play multifaceted roles in virus-host interaction and adaptation of the virus to the host^3–5^.

Complex glycans are added to viral surface proteins as a part of the post-translational modification during viral replication in the host. The adaptation of a virus to its host can involve mutations that either add or remove glycosylation sites so as to evade host immune response by either masking or modifying a previously exposed epitope surface^6–8^. Although complex glycans on the viral surface are not likely to elicit an immune response, the clustering of these glycosylation sites on the viral surface antigens can generate distinct types of glycans that are different from the typical glycans present on host glycoproteins^9^. Distinct high-mannose type N-linked glycan structures are present on clustered glycosylation sites of the gp120 envelope protein of the human immunodeficiency virus (HIV)^10^, primarily as a result of clustered N-linked glycans sterically-interfering with α-mannosidase function^11^. The presence of high-mannose N-linked glycosylation across diverse pathogen surfaces prompted previous studies to investigate if specific glycan-binding proteins or lectins can bind to these high mannose glycans and neutralize these viruses. Specifically, mannose-binding lectins including those from human C-type lectin family^12^ and plant lectins such as banana lectins^13^ have been demonstrated to bind high-mannose type glycan structures displayed on surfaces of diverse pathogens such as HIV and influenza viruses.

The distinct glycosylation patterns on viral surfaces also present “glycoepitopes” that are targets of antibodies generated by the host immune response to neutralize the virus. The evidence of such glycoepitopes is best established in the case of the HIV gp120^3,14^. Several antibodies isolated from HIV patients target these glycoepitopes on gp120 and effectively neutralize the virus^15–17^. Among these antibodies the 2G12 antibody is unique in two aspects. First, instead of the conventional “Y” shape with 2 antigen binding Fab domains (VH–VL, VH′–VL′), 2G12 has an additional binding site formed by cross over of VH domains of each Fab (VH–VL′, VH′–VL and VH–VH′)^18^. Second, unlike other HIV antibodies that interact with both the glycan and the underlying protein surface (“glycan-protein glycoepitopes”^19^), 2G12 recognizes a solely glycan-based glycoepitope comprised of high-mannose type glycans at glycosylation sites including N295, N332, N339, and N392 on gp120^20,21^. Mutational studies to knockout these four glycosylation sites and nearby sites on gp120 have provided insights on the relative contributions of each glycan to the binding affinity of 2G12^22^, revealing that 2G12 maintains high-affinity binding to a range of similar glycan configurations. Given this observation, in combination with evidence that 2G12 binds SARS-CoV-2 spike protein^23,24^, the hemagglutinin (HA) of certain Influenza H3N2 viruses^25^, and glycoproteins of *S. cerevisiae*^26^, the anti-glycan antibody 2G12 is likely to target the glycoproteins of additional viruses. Yet, as 2G12-glycoprotein interactions do not depend directly on the protein surface itself, existing methods to identify novel repurposing targets for antibodies based on protein sequences and structures are not suited for predicting new targets for 2G12.

In this study, we first present experimental data describing interactions between 2G12 and a set of H1N1 and H3N2 viruses. Our results are consistent with recently published data^25^ on 2G12-H3N2 interactions, yet extend these findings to certain H1N1 viruses and further demonstrate how certain N-glycan topologies on H3N2 enhance 2G12’s binding, functional inhibition, and neutralization potency. Second, we present a structural analysis of 2G12 interactions across HIV^22^, Flu^25^, and SARS-CoV-2^23^ to define a generalized model for 2G12 glycoepitopes based on N-glycan site topology at the protein surface. Finally, we hypothesized that any other viral surface glycoprotein that possesses a similar N-glycan site topology can potentially be recognized by 2G12. We therefore apply our model to survey the Protein Data Bank for glycoproteins that 2G12 putatively binds. The resulting “hits” serve as a list of viruses for which 2G12 repurposing toward analytical, diagnostic, and therapeutic applications may prove valuable.

## Results

### 2G12 binds, functionally inhibits, and neutralizes select Influenza H1N1 and H3N2

**Figure 1:**
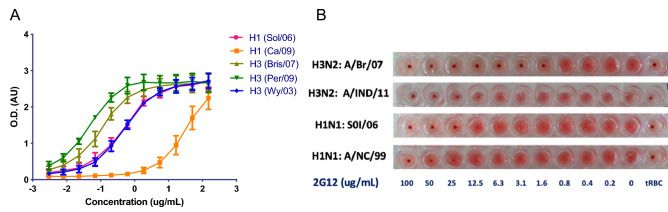
(**A**) Binding of 2G12 to full length recombinant HA from representative H1N1 and H3N2 strains in a quantitative ELISA (n = 3). Pink circles, HA from A/Solomon Island/2006 (H1N1); orange squares, HA from A/California/2009 (H1N1); dark green triangles, HA from A/Brisbane/2007 (H3N2); green triangles, HA from A/Perth/2009 (H3N2); blue diamond, HA from A/Wyoming/2003 (H3N2). (**B**) 2G12 inhibits agglutination of red blood cells by inactive influenza viruses. 4 HAU of inactive influenza viruses were incubated with serial dilution of 2G12 starting at 100 ug/mL for 45 min on ice followed by addition of 2% turkey red blood cells.

It was recently reported than 2G12 binds and neutralizes select H3N2 Influenza A viruses^25^. At the time of this publication, our lab was evaluating unpublished data for interactions between 2G12 and a number of Influenza A viruses which we report now to expand the understanding of these interactions. Here we provide evidence indicating that 2G12 binds, functionally inhibits, and neutralizes a number of additional Influenza A viruses including H1N1 lineages (Fig. 1, Table 1). Briefly, we find that 2G12 binds H1N1 A/SoI/06 and H3N2 A/Per/09, A/Bris/07, A/Wy/03 at concentrations below 1 ug/mL, but only very weakly binds H1N1 Ca/09 (Fig. 1A).

**Table 1:** 2G12 neutralizes select H3N2 viruses in vitro.

H3N2 influenza strains	Inoculum (PFUs)	EC50 (ug/mL)	CC50 (ug/mL)	SI
A/Brisbane/10/2007	40	0.1	> 100	> 10
A/Sydney/05/97	20	1.8	> 100	> 56
A/Perth/16/2009	32	1.5	> 100	> 67
A/Victoria/03/75	20	Not active	> 100	NA

EC50, CC50, and selective index (SI) are reported according to inhibition of CPE observed in MDCK cells.

Further, we evaluated 2G12’s ability to functionally inhibit the HA of certain representative H1N1 and H3N2 viruses. Turkey red blood cells (tRBCs) predominantly express α2 → 6 sialylated glycan receptors that specifically bind to HA from human-adapted viruses. We thus measured the ability of 2G12 to inhibit agglutination of tRBCs by representative human-adapted H1N1 and H3N2 viruses (Fig. 1B). We find functional inhibition of HA from both H3N2 and H1N1 viruses, with the strongest effect identified for H3N2 A/Br/07 (inhibition as low as 1.6 ug/mL). Inhibition of H1N1 viruses was weaker, with the strongest effect identified for A/SoI/06 (25 ug/mL).

Finally, consistent with 2G12’s effective inhibition of tRBC agglutination, we find that 2G12 neutralizes live H3N2 viruses. 2G12 potently inhibited the induction of CPE by Bris/07 with an EC50 of 0.1ug/ml. Inhibition of CPE induction by 2G12 was also observed for A/Sydney/97 (Syd/97) and A/Perth/11/2009 (Perth/09) H3N2 strains, albeit at a 10–20-fold lower concentration (Table 1). 2G12 failed to neutralize A/Vic/75. Importantly, the viruses surveyed here feature similar yet distinct N-glycans site topologies on the HA stalk, often in a ± 1 N-glycan site format. We therefore sought to evaluate the totality of the binding, inhibition, and neutralization data in light of the N-glycan topology for these viruses to expand our understanding of the structural basis for 2G12 interactions.

### A pair of N-glycan sites on the HA head is sufficient-but not optimal-for 2G12 interactions

Lee et al.^25^ reported that a pair of N-glycans on the H3N2 HA head at sites N165 and N246 mediate overhead 2G12 binding poses and are critical for 2G12 neutralization of H3N2 viruses. Our data expand the understanding of N-glycan topology required for 2G12 interactions with HA, providing evidence that (1) homologous pairs of N-glycans on the H1N1 head are sufficient to mediate 2G12 binding and functional inhibition, and (2) glycans other than N165 and N246 on the H3N2 head significantly enhance 2G12 binding, functional inhibition, and neutralization.

**Figure 2:**
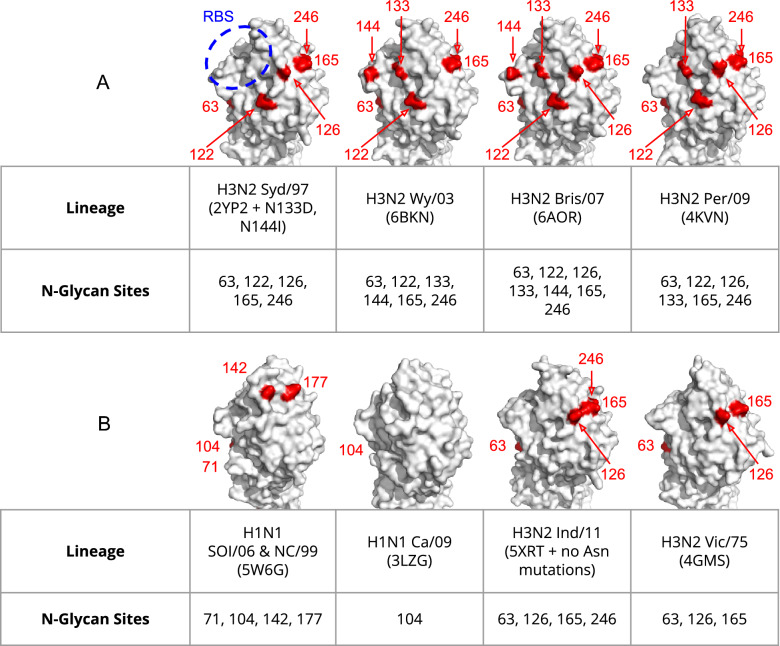
HA head N-glycan topology of Influenza A viruses tested for 2G12 binding, functional inhibition, and neutralization. Surface representations of HA are shown in white with N-glycan sites highlighted in red and the receptor binding site (RBS) outlined in blue. (**A**) A/Syd/97, A/Wy/03, A/Bris/07, and A/Per/09 all feature the N-glycan pair at N165 and N246, plus two to four additional adjacent N-glycan sites. These viruses are more potently bound, inhibited, and neutralized by 2G12, and include N-glycans with varying degrees of proximity to the RBS. (**B**) A/SoI/06, A/NC/99, A/Ind/11, and A/Vic/75 present pairs of N-glycan sites on the HA head, while A/Ca/09 features a single N-glycan site on the HA head (reverse, not shown). H1N1 and H3N2 viruses solely presenting N-glycan pairs on the HA head are weakly bound and functionally inhibited by 2G12 (Table S1).

First, we find that H1N1 A/SoI/06 and A/NC/99 present N-glycans pairs on the HA head (N142, N177; Fig. 2B), and that 2G12 binds and functionally inhibits these strains (Fig. 1A,B). In contrast, H1N1 A/Ca/09 presents a more substantial stalk N-glycan cluster than A/SoI/06 and lacks a pair of head N-glycans, but is only weakly bound by 2G12 at saturating concentrations. Together, these data suggest that the pair of head N-glycans on A/SoI/06 and A/NC/99 is responsible for 2G12 binding and functional inhibition of these viruses. These data highlight the critical role that a single pair of clustered N-glycans can play at quaternary junctions, and we hypothesize that the mode of interaction between 2G12 and A/SoI/06 is likely analogous to the overhead quaternary pose reported for H3N2 viruses by Lee et al.^25^.

Second, our data suggests that glycan pairs on the HA head alone are not optimal for 2G12 interactions. Examining the N-glycan site topology for H3N2 A/Ind/11 and H3N2 A/Vic/75 (Fig. 2A), which both feature N-glycan pairs in isolation, we find that these H3N2 viruses as well as the previously mentioned H1N1 viruses show weak functional inhibition (A/Ind/11 at 12.5 ug/mL, A/SoI/06 at 25 ug/mL, A/NC/99 at 25 ug/mL) and a lack of neutralization (no neutralization observed for A/Vic/75) relative to Flu viruses presenting additional head N-glycans. That is, H3N2 viruses bearing more substantial N-glycan clusters are the most potent binders (A/Per/09 and A/Br/07), inhibitors (A/Br/07 at 1.6 ug/mL), and neutralizers (A/Br/07 EC50 at 0.1 ug/mL). Note that we find 2G12 binding strength is not directly correlated with the number of head N-glycans, suggesting the specific N-glycan cluster topology influences 2G12 activity. While N-glycan pairs may be sufficient—and certain N-glycan pairs may be critical—these pairs alone do not appear optimal for 2G12 interactions.

The structural data on Flu-2G12 interactions presented by Lee et al.^25^ suggests that non-overhead or partially-overhead binding poses may explain how additional head N-glycans optimize 2G12 binding and neutralization. The Lee et al.^25^ negative-stain electron microscopy (nsEM) images indicate a variety of poses for 2G12 bound to HA that include poses in which 2G12 is not strictly overhead of HA and others in which 2G12 runs parallel to the vertical axis of HA^25^. These additional poses suggest that N-glycans other than N165 and N246 may be directly involved in the 2G12 binding event. We therefore next examine how different head N-glycan topologies contribute functionally to 2G12-Flu interactions.

### There is a binding vs. neutralization tradeoff dictated by N-glycan topology on the H3N2 HA head

While it is clear that additional N-glycan clustering on the HA head contributes to more effective 2G12 interactions, our data provide further evidence that the specific topology of head glycans drives a tradeoff between binding strength and neutralization potency. We observe that the strongest binder (A/Per/09, EC50 = 0.35 nM) is not the most potent neutralizer (EC50 = 1.5 ug/mL). Rather, A/Bris/07 neutralizes with more than an order of magnitude higher potency (EC50 = 0.1 ug/mL) despite binding with twofold lower strength (EC50 = 0.73 nM). The key difference between the N-glycan topologies for these two viruses is the additional N-glycan at site N144 for A/Bris/07. The N144 N-glycan is directly adjacent to the major functional site for HA—the receptor binding site (RBS)—offering a straightforward explanation for the significant increase in neutralization potency against A/Bris/07. That is, the N144 glycan may promote 2G12 binding poses that sterically interfere with RBS function to a greater extent than HA glycan clusters lacking an N144 glycan. Further, it is plausible that binding strength to A/Bris/07 is relatively reduced as a result of the glycan at site N133 disrupting binding poses spanning from N144 to N165/N246, as the N133 glycan would reside in the middle of the 2G12 paratope potentially creating a steric constraint for 2G12 binding. Such a mechanism has precedent in the literature on interactions between 2G12 and gp120 in which the glycan at site N411 resides in the middle of the 2G12 paratope, and it has been observed that knockout of this glycan enhances 2G12 binding strength beyond that of wild type gp120^22^. Indeed, consistent with these two hypotheses, the highest affinity monomeric glycoepitope (A/Per/09) is highly planar and can be visualized as presenting both monomeric and quaternary glycoepitopes according to the poses imaged in Lee at al.^25^. We next sought to integrate these functional observations across H1N1 and H3N2 viruses alongside existing structural models of 2G12 interactions with HIV and SARS-CoV-2 to build a topological model for 2G12 interactions with predictive potential.

### Model of 2G12 topological glycoepitopes across HIV, Flu, and SARS-CoV-2

**Figure 3:**
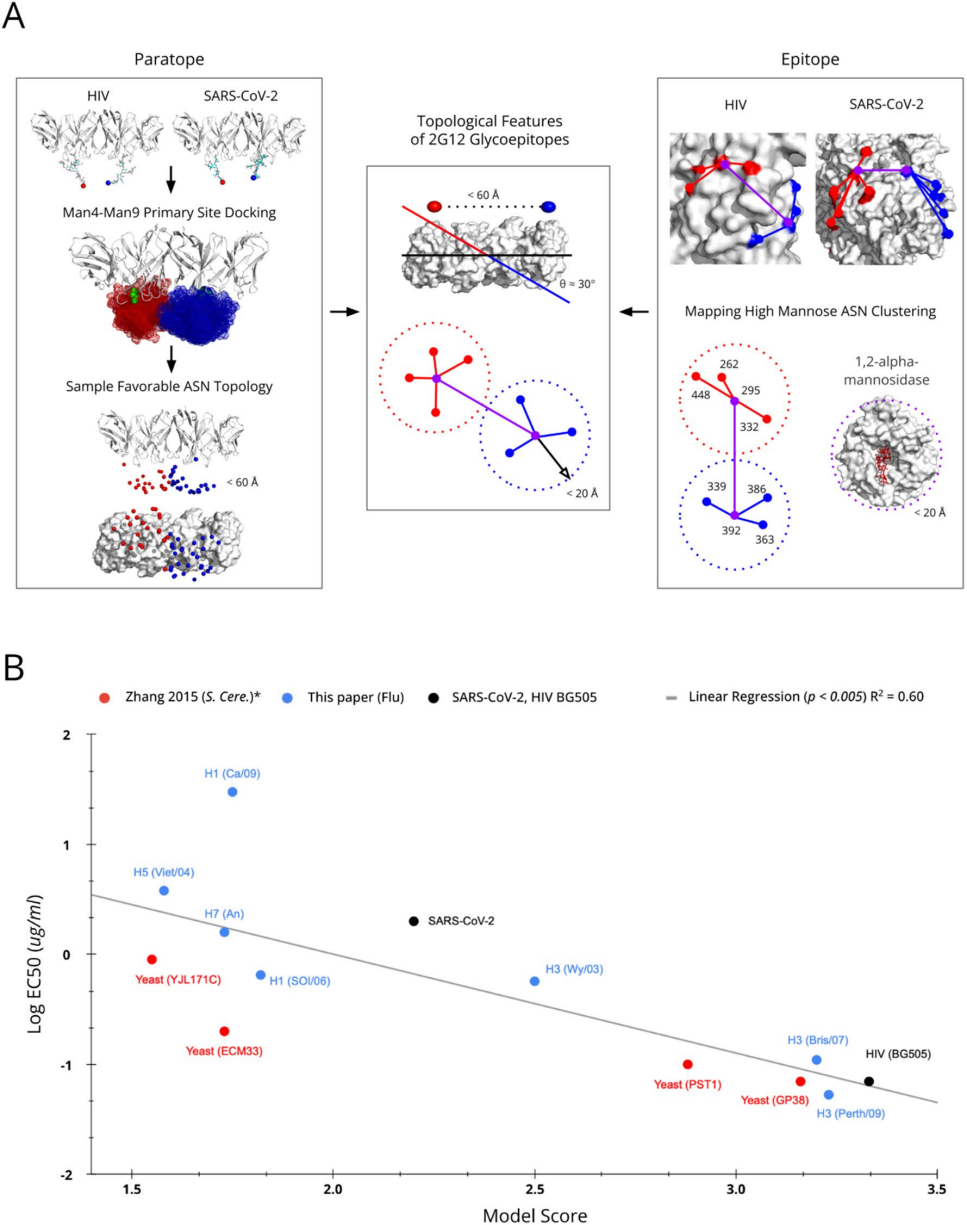
2G12 Topological glycoepitope model and validation. (**A**) Left, Paratope: From the structures of 2G12 complexed to HIV BG505 (6OZC^22^) and SARS-CoV-2 spike (7L09^23^), we derive a distribution of canonical Man4–Man9 poses (shown as mesh) amenable to binding the 2G12 primary binding sites, and a corresponding distribution of N-glycan sites (shown as individual spheres). The most distant pair of N-glycan sites giving rise to a theoretically acceptable 2G12 binding pose are 60 Å apart. Right, Epitope: From the epitope side of the 2G12 interactions, we examine the clustering of primary (purple spheres) and secondary (red or blue spheres) N-glycan sites, and the steric constraints of ER α1,2-mannosidase^11^ (5KIJ^29^). These features contribute to oligomannosylation at the primary Asn, secondary binding site interactions, and indirectly to the binding event via network effects (e.g., glycan support and stability). Middle: We integrate information from the epitope and paratope sides of the interaction to develop a simple feature set describing N-glycan site topologies likely to present 2G12 glycoepitopes. (**B**) 2G12 glycoepitope scores correlate with 2G12 binding strength. The apparent binding strength for 2G12 and 13 viral and non-viral glycoproteins measured in this study and elsewhere is plotted against the 2G12 glycoepitope score returned by the glycoepitope model for each glycoprotein. A statistically significant linear relationship is observed (*p* < 0.005), suggesting that the glycoepitope model provides a degree of predictive utility. *Note that for the glycoproteins measured in Zhang et al., 2015^26^, solved structures do not exist and so high confidence Alpha Fold 2^28^ predictions were employed (90%+ predicted accuracy for all sites). The *S. cerevisiae* glycoprotein predictions are limited to monomeric glycoepitopes, potentially explaining their systemic underscoring relative to other glycoprotein structures scored that include both monomeric and quaternary glycoepitopes.

To define a generalizable model for topological glycoepitopes recognized by 2G12, we leveraged data across the three known viruses 2G12 binds to: HIV, Flu, and SARS-CoV-2. Specifically, we analyzed structural features from co-crystal complexes (6OZC^22^; 7L09^23^) and functional data from experimental binding and neutralization assays (38; Fig. 1). Further, we incorporated the theoretical basis for clustered N-glycans promoting high-mannose species^11^ and performed computational glycan docking experiments to derive broad distributions of allowable 2G12 binding poses. By integrating these data, we learn from both the epitope and paratope sides of the 2G12 epitope-paratope interaction to define a generalizable topological model (Fig. 3).

As observed in the structure of 2G12 bound to HIV BG505 SOSIP.664 (6OZC^22^), 2G12 has two distinct primary binding sites with each site binding to a high mannose glycan at N295 and N392 with preference for the terminal α1–2 linked-mannose at the d1 arm of Man9^18^. Further, 2G12 presents a third binding “surface” formed at the VH–VH′ crossover interface, which interacts with additional mannose sugars in the d2 and d3 branches of two high-mannose glycans at N332 and N339^22^. Meanwhile, the 2G12-SARS-CoV-2 spike complex (7L09^23^) indicates that a single high-mannose N-glycan at N717 is capable of interacting with both the primary and secondary 2G12 binding sites simultaneously. Data from 2G12-H3N2/H1N1 interactions (38, Fig. 1) suggest that a single pair of high-mannose N-glycans is capable of meditating 2G12 binding via the primary and secondary binding sites, though there is insufficient structural data to determine the nature of the secondary interactions.

Considering the totality of this structural and functional data it is appealing and logical to define a glycoepitope model based on N-linked glycans whose terminal mannose form a rhombus shape mirroring the 2G12 paratope, with the long axis defined by the primary binding sites (VH–VL′, VH′–VL) and the short axis defined by the secondary surface (VH–VH′). However, such a definition would limit application of the model as it would depend critically on N-glycan structures which tend not to be fully solved in most glycoprotein structures. Further, even structures that include full-length high-quality N-glycans (or computational approaches to populating N-glycans) would still not represent the range of conformations flexible glycans can adopt during 2G12 binding. Thus, we instead build our model based on the topology of Asn residues at N-glycan sites, as the minimal binding determinant for 2G12 appears to be a pair of N-glycan sites presenting high-mannose N-glycans capable of interacting with the two 2G12 primary binding sites, while nearby N-glycans sites clustered around these two primary N-glycan sites promote high-mannose species at the primary sites and interact with the secondary VH–VH′ binding surface. Toward not overfitting the limited structural information available (i.e., scoring glycoepitopes based on an “ideal” distance between primary binding N-glycan sites for gp120 of ~ 21.9 Å), we selected the simplest feature set that satisfies these objectives while also modeling distributions of possible N-glycan poses (Fig. 3), specifically:

Feature 1: A surface exposed pair of N-glycan sites, wherein the two Asn residues (termed *primary Asn*) can be docked with canonical Man4-Man9 glycans such that there is a pose in which both glycans interact via a terminal mannose with the 2G12 primary binding sites.
Feature 2: Additional surface exposed N-glycan sites (termed *secondary Asn*) whose Asn residue is sufficiently close to a *primary Asn* to (1) sterically impair N-glycan processing by α1,2-mannosidase and (2) interact with the secondary binding surfaces of 2G12, where the number and proximity of these secondary sites is proportional to the likelihood of mediating these effects.

We derive primary Asn site distributions from a glycan docking exercise using 25 canonical Man4-Man9 glycans obtained from the Glycan Fragment Database^27^, and clustering radii are computed based on steric constraints of ER α1,2-mannosidase^11^ (Fig. 3A; see “Materials and methods” section). From this derivation, we find that satisfactory primary Asn pairs lie along a 60 Å axis that is rotated approximately 30° relative to the 2G12 paratope, and that secondary Asn tend to reside within 20 Å of each primary Asn. We subsequently wrote a lightweight algorithm to score all combinations of N-glycan sites on a given glycoprotein structure based on these two features (see “Materials and methods” section).

Toward validating the 2G12 glycoepitope model, we examined the relationship between model score and apparent 2G12 binding strength to 13 viral and non-viral glycoproteins (Fig. 3B). We identified 13 glycoproteins with measured 2G12 binding strength in the literature or in our lab, consisting of the following antigens: HIV-derived BG505^22^, SARS-CoV-2 spike protein^23^, Hemagglutinin from seven Influenza lineages spanning four sub-types, and four non-viral glycoproteins derived from *S. cerevisiae*^26^. Structures of the four *S. cerevisiae* glycoproteins are not solved and so were predicted via Alpha Fold 2^28^, which returned predicted structures with high confidence (90%+) at all sites. We found a linear correlation between the apparent 2G12 binding strength and the modeled glycoepitope score (R^2^ = 0.60, *p* < 0.005), suggesting that the model offers predictive value for identifying high-affinity 2G12 glycoepitopes. Notably, the *S. cerevisiae* glycoproteins appear to be systematically underscored by the algorithm. One potential explanation for this divergence from the trend is that the *S. cerevisiae* glycoepitopes are scored based solely on monomeric presentation. That is, the 2G12 binding experiments were performed under conditions in which quaternary interactions may occur, but the *S. cerevisiae* glycoproteins are scored by the model according to monomeric glycoepitope presentation because no structures of these glycoproteins exist in the proper quaternary biological context. Having defined a model for scoring 2G12 glycoepitopes based on N-glycan site topology, we next apply it to identify viruses with putative 2G12 glycoepitopes in the Protein Data Bank.

### Predicting putative 2G12 glycoepitopes on novel viruses

**Figure 4:**
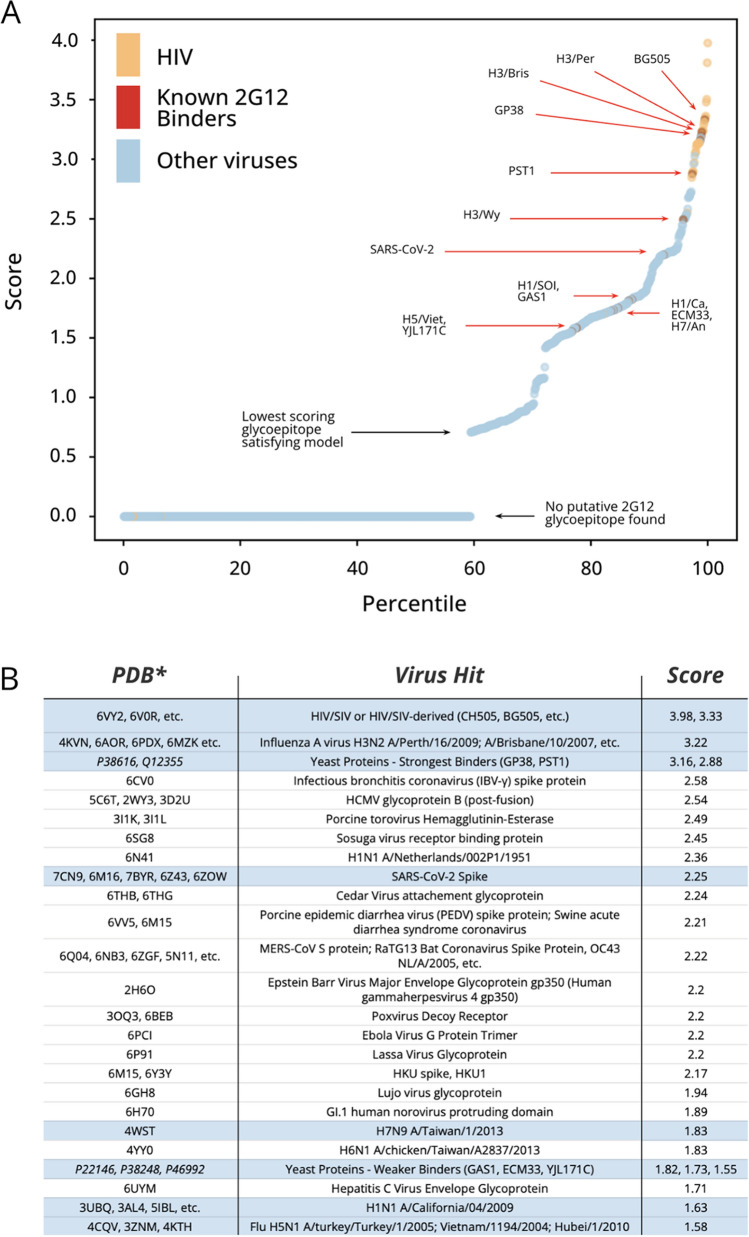

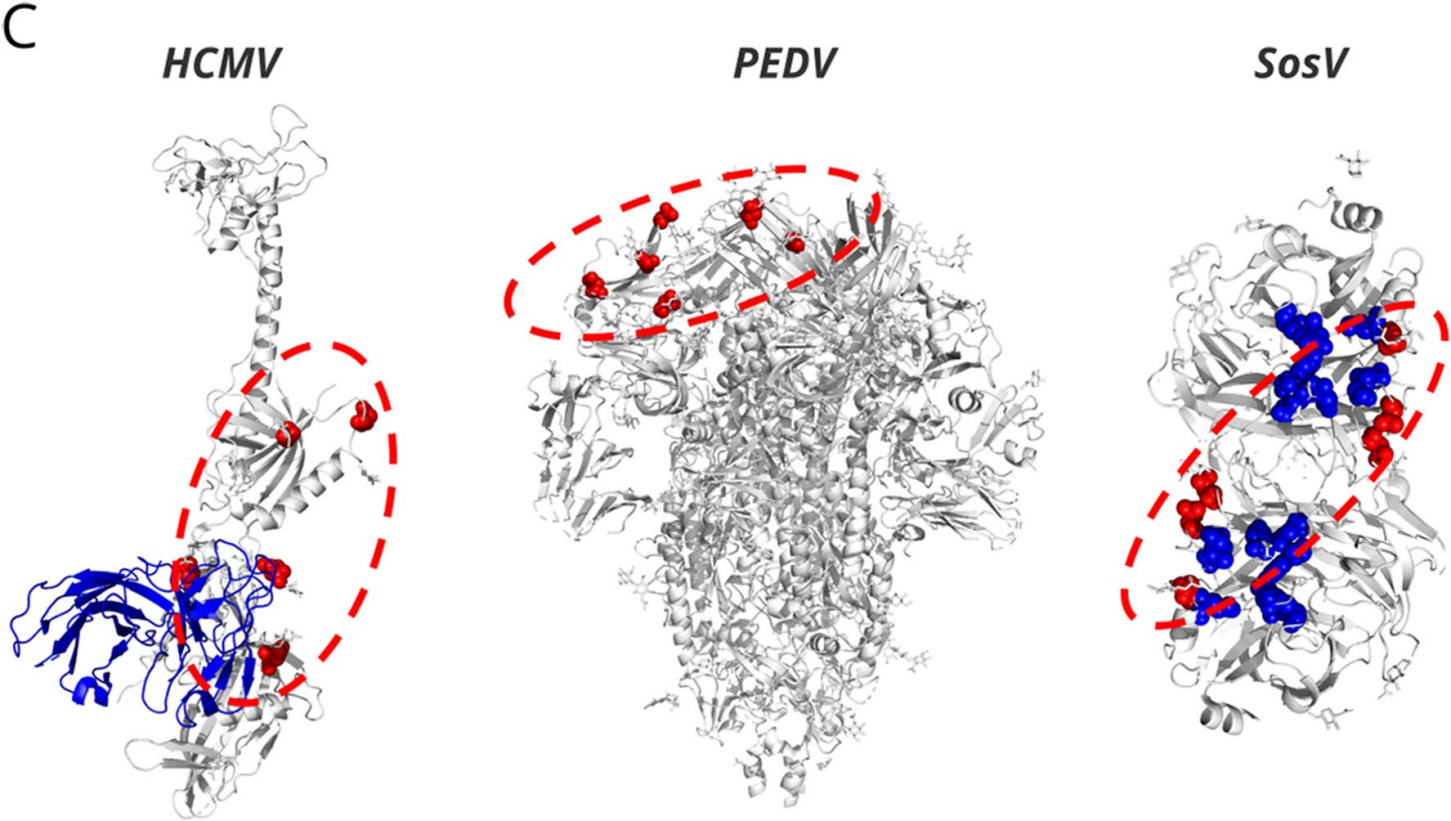
PDB search for 2G12 repurposing targets. (**A**) Distribution of 2G12 glycoepitope scores for 1250 + viral glycoproteins in PDB. Each structure analyzed is plotted according to its top scoring 2G12 glycoepitope and ranked against all other glycoproteins in the set. Approximately 60% of viral glycoproteins did not present an N-glycan topology satisfying the minimum criteria of the model. The weakest known 2G12 binding glycoproteins score in the 80th to 90th percentiles and the top scoring glycoproteins are near exclusively HIV/SIV or HIV-derived glycoproteins (e.g., BG505). (**B**) Top scoring viral glycoproteins. Rows corresponding to known 2G12 binders are highlighted in light blue. *For *S. cerevisiae* glycoproteins, UniProt accession codes are shown. (**C**) Three hits with logical mechanisms of neutralization are shown, with the Asn residues for the highest scoring glycoepitope for each glycoprotein shown as red spheres and the putative 2G12 footprint shown as a red dotted oval. Left, the post-fusion glycoprotein B of HCMV features a 96th percentile scoring glycoepitope for which half of the glycoepitope footprint overlaps with a known neutralizing epitope (PDB: 5C6T^30^, nAb 1G2 shown in blue). Note that the pre-fusion conformation also presents a putative, albeit lower scoring, 2G12 glycoepitope. Middle, the spike protein of PEDV features a number of high-scoring glycoepitopes (96th percentile) that suggest inter- and intra-chain locking (PDB: 6VV5^31^). Right, the receptor binding protein of Sosuga Virus (SosV) features a glycoepitope scoring in the 95th percentile that overlaps with the expected receptor binding site, which is shown in blue (PDB: 6SG8^32^).

Finally, we sought to apply our 2G12 glycoepitope model to search for additional viruses that might be bound by 2G12 toward repurposing 2G12 for analytical, diagnostic, or therapeutic purposes. We identified over 1250 viral glycoproteins in the Protein Data Bank and scored them using our 2G12 glycoepitope model (Fig. 4). We obtained a score-percentile distribution in which approximately 40% of viral glycoproteins in the set display a pair of N-glycan sites satisfying the minimal topological constraints for presenting a potential 2G12 glycoepitope if both of these N-glycan sites were occupied by high-mannose N-glycans (Fig. 4A). However, it is unlikely that 2G12 binds these lowest scoring glycoproteins as the lowest scoring glycoproteins do not offer sufficient secondary N-glycan sites to promote high-mannose species at the primary sites or to interact with the secondary 2G12 binding surface. Rather, the lowest experimentally-verified 2G12 binders occur at the 80th score percentile (H5/Viet/04 and YJL171C) and the strongest known binders (EC50 ≤ 0.1 ug/mL) score in the 95th percentile or higher. Further, the 98th and above percentiles contain near exclusively HIV/SIV Env and HIV/SIV constructs (e.g., BG505 and CH505) reflecting the unique degree of N-glycan clustering within the HIV intrinsic mannose patch.

We therefore defined a set of “hits” as all glycoproteins with scores above the lowest scoring known 2G12 binder (H5/Viet/04) for manual investigation of structures presenting 2G12 glycoepitopes in a plausible biological context (Fig. 4B). During this curation process we eliminated enzymes, non-structural proteins, and glycoepitopes presented proximal to membranes or multimeric interfaces such that 2G12 is unlikely to bind in the context of the full virion. We further moved structures of viruses that do not infect eukaryotic cells and those containing multiple copies of glycoproteins in a non-biologically relevant conformation (e.g., in the crystal lattice). For a number of these hits, including HCMV glycoprotein B, porcine epidemic diarrhea virus (PEDV) spike protein, and Sosuga virus (SosV) receptor binding protein, the predicted 2G12 glycoepitope overlaps known neutralizing antibody epitopes or functionally critical domains and therefore presents a plausible basis for 2G12 neutralization (Fig. 4C). Future investigation is warranted to determine if 2G12 offers diagnostic, analytical, or therapeutic utility for these viruses presenting putative 2G12 glycoepitopes.

## Discussion

2G12 was one of the first identified broadly neutralizing human antibodies directed against the high-mannose glycans of HIV gp120^33,34^. The unique structure of 2G12 presenting three distinct high-mannose glycan binding surfaces was subsequently described^15,18,35^. Following recent reports that 2G12 interacts with Influenza H3N2 and SARS-CoV-2, we report results demonstrating that 2G12 binds and functionally inhibits select Influenza A H1N1 viruses and additional H3N2 viruses. Through follow-up work to validate our algorithm, we further observed 2G12 binding to H5 and H7 strains. Further, our results indicate that pairs of N-glycans on the HA head are sufficient to mediate 2G12-HA interactions with H1 HA, which is consistent with the critical role identified by Lee et al., for N165 and N246^25^ on H3 HA. Yet, our results also indicate that additional glycans beyond a sole pair substantially enhance 2G12 interactions and contribute to more potent neutralization.

Additional N-glycans may mediate enhanced effects through a variety of direct and indirect mechanisms. Potential direct effects include glycans proximal to the HA receptor binding site (RBS) interacting directly with the 2G12 paratope, which may favor 2G12 poses that sterically obstruct HA function. Indeed, a variety of 2G12 poses for a given HA are observed by Lee et al.^25^, suggesting certain N-glycan topologies may more strongly favor certain overhead vs. RBS-proximal poses. On the other hand, the additional N-glycans likely exert an indirect functional effect by modulating glycosylation state at the primary binding site glycans or contributing to glycan network effects such as stabilization. Indeed, Lee et al., present evidence that N165 and N246 are nearly entirely Man9 species, but it was previously unclear whether this effect is dependent on the other H3N2 head glycans or if the pair is sufficiently clustered to induce Man9 alone. Our results demonstrating 2G12 binding to a pair of N-glycans on the HA head of select H1N1 viruses suggest that a lone pair is indeed sufficient to induce high-mannose N-glycosylation and bind 2G12. Still, 2G12 is capable of binding a variety of Man4+ species^21^, and so our H1N1 results alone do not indicate whether the pair promotes Man9 or other Man4+ species, leaving open the possibility that additional N-glycans on the H3N2 head affect the high-mannose species present at the critical binding pair. Our results further suggest that subtle differences in HA head glycan topology at sites beyond the N165/N246 pair produce substantial effects including a tradeoff between binding and neutralization for H3N2 viruses.

Our study has implications in two specific areas. *First*, our findings have direct relevance to influenza research and drug development efforts. Since inception into human populations, influenza viruses have acquired mutations that add or subtract glycosylation sites, with additions occurring in intervals of about 5 to 7 years^36^. Interestingly, the introduction of the swine origin 2009 H1N1 virus as the dominant seasonal strain reset the clock on glycosylation sites in the RBS of HA in H1N1 viruses. Compared to previous seasonal strains the 2009 H1N1 lacked glycosylation sites in the RBS of HA resulting in the unmasking of novel antigenic epitopes^37^. In contrast, despite the identification of triple reassortant H3N2 viruses lacking glycosylation sites in the RBS of HA, these strains haven’t replaced the seasonal virus as the dominant strain and hence the glycosylation clock hasn’t been reset for H3N2 viruses yet. Thus, the HA from seasonal H3N2 viruses since 1968 until 2011 has been accumulating glycosylation sites in the RBS. A recent glycomic analysis demonstrated that these high-mannose N-glycan sites contribute to pathogenicity^38^. Based on this work, 2G12 may have utility as a diagnostic and analytical tool to monitor the evolution of glycosylation sites in circulating H3N2 viruses. Additionally, given the high neutralization potency of 2G12 against select H3N2 strains, 2G12 might prove useful as a therapeutic. Further, 2G12 should be considered if there is ever a need for a rapid response against an emerging strain with novel N-glycosylation sites proximal to the RBS as 2G12 may potently neutralize such a strain.

*Second*, our approach to define glycoepitope similarity based on structural and geometric considerations goes beyond the conventional definition based on protein sequence similarity and permits identification of novel target epitope surfaces that can be successfully targeted by anti-glycan antibodies on diverse antigens. While until recently 2G12 was the only known antibody recognizing solely N-glycan-based epitopes, an additional structural class of antibodies known as Fab-dimerized glycan-reactive (FDG) antibodies was recently reported^39^. Further, the set of mutations required to mediate VH domain exchange on the 2G12 scaffold is known^40^, suggesting the possibility of converting conventional antibodies with glycan-binding domains (e.g., the PGT series^16^) to domain-exchanged format, potentially mediating enhanced glycan-specific binding. Converted antibodies could be subsequently optimized for interactions with glycoepitopes bearing other glycan species or topologies than those preferred by 2G12 or the FDG antibodies documented to date. Therefore, the approach presented in this study may generalize to guide repurposing of additional anti-glycan antibodies to target topologically similar glycoepitope surfaces on diverse antigens. Further, given recent advances in predicting protein structure from sequence alone^41,28^ and the application of Alpha Fold 2 to predict and score the structure of *S. cerevisiae* glycoproteins in this paper which correlated well with measured binding strength, the 2G12 topological model could also be applied to identify novel viral glycoproteins targets for 2G12 from the vast sequence space of emerging viruses.

## Materials and methods

### Antibody expression and purification

2G12 variable heavy and light chain were cloned into full length IgG1 expression vector pcDNA3.3 HC and pcDNA3.3 LC. The antibodies were transiently expressed in Freestyle 293 expression system (Invitrogen, CA) and purified using Protein A Sepharose affinity chromatography (Pierce Biotech, GE, UK). The purified antibodies were concentrated, and buffer exchanged into 1× PBS using 30 kDa spin filter columns (Milipore, USA) and quantified using a nanodrop.

### Enzyme linked immunoabsorbent assay (ELISA)

For ELISA, Maxisorp high-binding 96 well microtiter plates were used to coat the antigen. All steps of the ELISA were performed at room temperature and all volumes used were 100 uL unless otherwise stated. Wells were coated with 50 ng of gp120 JR-FL or 200 ng/well of the various full length HA trimer overnight at 4 °C. Plates were washed three times with 1× PBS/0.05% (vol/vol) Tween 20, blocked for 1 h with 5% Blotto/PBS/0.05% Tween 20 and washed three times with PBS/0.05% Tween 20. Serial dilutions of all antibodies in PBS/0.05% Tween 20 were incubated for 2 h, and the unbound antibody was removed by washing three times as described above. The binding was detected via rabbit anti-human Fcγ HRP conjugate (1:10,000, Jackson Immuno Research, PA) and TMB substrate at 405 nm.

### Viruses

The following inactive viruses were used in the study. A/Solomon Island/2006 (H1N1) (Meridien Life Sciences), A/New Caledonia/99 (H1N1) (Fitzgerald), and A/Brisbane/2007 (H3N2) (Meridien Life Sciences).

### Hemagglutination inhibition assay

Hemagglutination inhibition assays were performed using turkey red blood cells (tRBC; Rockland, cat: R408-0050). Briefly, the pooled and washed 10% tRBC were centrifuged at 1000 rpm to pellet the cells. The supernatant was removed, and the pellet was resuspended in 1× PBS. This process was repeated three times and the cells were finally resuspended in 1× PBS to give a final concentration of 2% red blood cells. Twofold serial dilution of the different viruses was incubated with an equal volume of 2% tRBC to determine the HA units for each of the viruses. For the hemagglutination inhibition assay, 4-HA units of each virus was pre-incubated on ice for 45 min with serial dilution of the various antibodies. At the end of the incubation, an equal volume of 2% tRBC was added and incubated on ice for an additional 1 h.

### In vitro neutralization against select strains

2G12 was evaluated against a select set of H3N2 virus strains via a standardized cytopathic effect (CPE) inhibition assay at The Institute for Antiviral Research (IAR) at Utah State University. Briefly, 8 serial dilutions of 2G12 were added to MDCK cells (IAR) infected with the specific H3N2 viruses. PFUs were measured according to cell culture infective dose (CCID50). The ability of 2G12 to prevent formation of CPE was measured 7 days post infection by neutral red uptake assay. In parallel, the toxicity was also determined and the selectivity index (SI) against each virus was calculated.

### Statistical analyses

The following statistical analyses were used in this study. Figure 1: A nonlinear log(agonist) vs. response curve with four parameters was fit to the observations. The error bars represent standard error of the mean (SEM) across three replicates. The R^2^ describing goodness of fit is > 0.90 for all curves. Figure 3: Linear regression was performed via the SciPy^42^ linregress function in Python. The linear regression was evaluated for statistical significance using a Wald test with t-distribution. The null hypothesis was defined as a slope of zero.

### 2G12 glycoepitope-paratope analysis

The 2G12 glycoepitope epitope-paratope analysis was carried out using models of 2G12 bound to HIV (6OZC^22^) and SARS-CoV-2 (7L09^23^). Epitope**:** For each model, 5 canonical N-glycans for each of man4-man9 (from the Glycan Fragment Database^27^) were docked into each primary binding site using the terminal mannose for alignment. The distribution of possible N-glycan sites giving rise to the array of N-glycan poses was estimated from the position of the terminal GlcNAc. Paratope: For each model, the N-glycan sites interacting with the 2G12 paratope were visualized and annotated in PyMol^43^ which were then used to construct network models toward building intuition for feature definition. To define the clustering radius of inclusion for secondary N-glycan sites, we analyzed the surface of ER α1,2-mannosidase adjacent to the enzymatic pocket as previously proposed^11^ (5KIJ^29^). Validation: Apparent binding constants were estimated according to EC50 for HIV^22^, SARS-CoV-2^23^, Flu (this study), and *S. cerevisiae*^26^ glycoproteins. For H1 and H3 lineages, EC50’s from Fig. 1 were used. For H5 and H7 lineages, EC50s using ELISA as decribed above but with a single replicate (data not shown). For scoring *S. cerevisiae* glycoproteins, high confidence (90%+) Alpha Fold 2^28^ predicted structures were employed according to Uniprot accession codes P38616, Q12355, P22146, P38248, and P46992. For the other glycoproteins, scores were computed on the following structures: 6AOR^44^, 4KVN^45^, 6BKN^46^, 3LZG^47^, 5UJZ^48^, 6V0R^49^, 6VXX^50^, 3ZNM^51^, 4LKI^52^. Note that the biological assembly was used to populate trimeric HA in cases where the given structure was monomeric. Linear regression was performed via the SciPy^42^ linregress function in Python.

### 2G12 glycoepitope search

The Protein Data Bank was first searched for structures annotated as derived from viruses and containing at least three putative N-glycan sites, and all hits were downloaded. For each structure, surface exposure was calculated for all N-glycan sites using a relative surface area (RSA) of 0.2 via the FreeSASA library^53^ and the theoretical MaxSASA values derived by Tien et al.^54^. For each surface exposed N-glycan site, the structure was searched for all other surface-exposed N-glycan sites within 60 Å (Feature 1). For each identified pair, all surface-exposed N-glycan sites whose ND2 atoms are within 20 Å of the ND2 atom of a pair-residue were identified and assigned to the nearest pair-residue (Feature 2). Secondary N-glycan sites were weighted from 0.5 to 1 depending on distance from the primary site (Feature 2). Finally, for each structure all glycoepitopes satisfying the minimal constraints were scored and ranked according to the sum of the square roots of the total weighting for both primary binding clusters. Top hits were manually curated for high relevance and feasible biological context.

## Supplementary Information

Supplementary Table S1.
