# Supplementary material for: Conserved topology of virus glycoepitopes presents novel targets for repurposing HIV antibody 2G12
Source: Sci Rep. 2022 Feb 16;12:2594. doi: 10.1038/s41598-022-06157-z (PMC8850445; doi:10.1038/s41598-022-06157-z)
Supplement: Supplementary file 1 — Supplementary Table S1. [file 41598_2022_6157_MOESM1_ESM.docx]

**Table S1.** Influenza strains and corresponding PDB structures used across experimental and structural analyses.

| **Influenza Strains** | **PDB Used:** resolution (identity/similarity/glycan site differences if different strain) |
| --- | --- |
| **H1N1** | |
| A/New Caledonia/1999 | **5W6G** (96.3% / 98.5% / none) |
| A/Solomon Island/2006 | **5W6G** [monomer, 2.79Å], **5UJZ** [trimer, 4.8Å] |
| A/California/2009 | **3LZG**: 2.6Å |
| **H3N2** | |
| A/Victoria/03/75 | **4GMS**: 2.95Å |
| A/Sydney/05/97 | **2YP2**: (92.4% / 96.0% / D133N, I144N) 1.9Å |
| A/Wyoming/03/2003 | **6BKN**: 1.85Å |
| A/Brisbane/10/2007 | **6AOR**: 1.7Å |
| A/Perth/16/2009 | **4KVN**: 3.1Å |
| A/Indiana/2011 | **5XRT**: (100% / 100% / none) 3.15Å |
